# Ernst von Leyden (1832–1910): a pioneer in making oncology a respected medical discipline

**DOI:** 10.1007/s00432-021-03746-9

**Published:** 2021-07-21

**Authors:** Peter Voswinckel, Nils Hansson

**Affiliations:** 1 German Society of Haematology and Oncology [DGHO], Berlin, Germany; 2 Department for the History, Philosophy, and Ethics of Medicine, Heinrich-Heine-University Duesseldorf, Moorenstr. 5, 40225 Duesseldorf, Germany

**Keywords:** History of cancer, Genesis of oncology, Internationality, Antisemitism, Ernst von Leyden

## Abstract

**Purpose:**

This article presents new research on the role of the renowned German physician Ernst von Leyden (1832–1910) in the emergence of oncology as a scientific discipline.

**Methods:**

The article draws on archival sources from the archive of the German Society of Haematology and primary and secondary literature.

**Results:**

Leyden initiated two important events in the early history of oncology: the first international cancer conference, which took place in Heidelberg, Germany, in 1906, and the founding of the first international association for cancer research (forerunner of today's UICC) in Berlin in 1908. Unfortunately, these facts are not mentioned in the most recent accounts. Both had a strong impact on the professionalization of oncology as a discipline in its own right.

**Conclusion:**

Although not of Jewish origin, von Leyden was considered by the National Socialists to be “Jewish tainted”, which had a lasting effect on his perception at home and abroad.

## First International Cancer Congress 1906

**Fig. 1:**
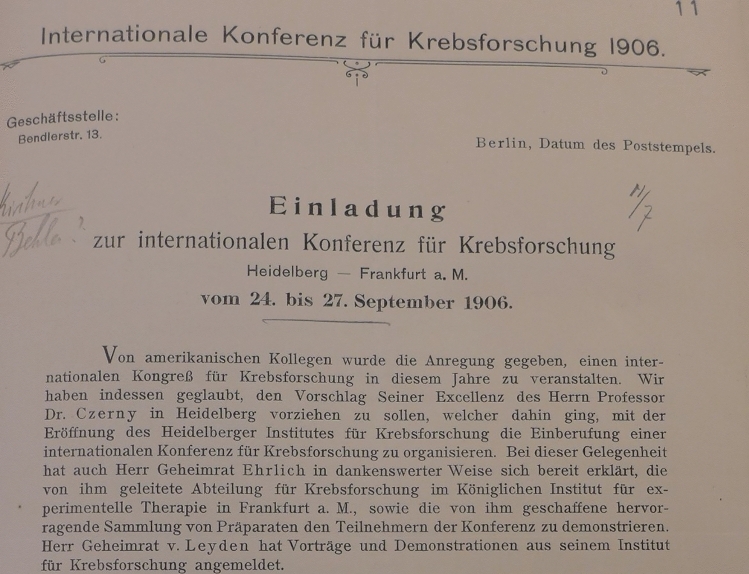
Letter of invitation to the first International Conference on Cancer Research in 1906. (Clipping) Secret State Archives Berlin (GStA PK). "American colleagues made the suggestion of holding an international congress on cancer research this year. However, we agreed with the suggestion of His Excellency Professor Dr. Czerny, Heidelberg, to select Heidelberg as a preferable location for this congress because it would coincide with the opening of the Heidelberg Institute for Cancer Research. Professor Dr. Czerny then went about calling for the organization of the international conference for cancer research. On this occasion, Mr. Privy Councilor Ehrlich also graciously declared his willingness to show the Department of Cancer Research which he heads at the Royal Institute for Experimental Therapy in Frankfurt am Main and to demonstrate the outstanding collection of specimens he created to the conference participants."

On June 13, 1906, American newspaper readers learned from the Los Angeles Times that the first international conference on cancer research ever was to be held in Germany. The previous day, Reuters had circulated the announcement all over the world; in addition, 900 personal letters of invitation had been sent out from Berlin to German and foreign doctors, some of whom only tangentially involved in cancer research. The invitation began with the preamble “American colleagues made the suggestion…”(Voswinckel 2019). (Fig. 1) The letter was signed by Ernst von Leyden (Berlin), Vincenz Czerny (Heidelberg) and Paul Ehrlich (Frankfurt). It was sent from the Berlin address of George Meyer, then Secretary General of the German Central Committee for Cancer Research (founded in 1900 by Ernst von Leyden).

“The opening of the First International Cancer Congress in Heidelberg was an event in the scientific world”—recalled 30 years later Ferdinand Blumenthal, doyen of German cancer research and student of Leyden (Blumenthal 1936). The signal effect that emanated from this event is today largely forgotten and urgently needs reconsideration. Newly discovered documents underline the great importance of the 1906 Heidelberg Conference to the institutionalization of international cooperation and scientific exchange between oncologists. On the day of the opening ceremony (September 25, 1906) the New York Tribune reported how Leyden had “[…] emphasized the necessity of the cooperation of all nations and all specialists in the struggle against the terrible suffering resulting from cancer” (New York Tribune Sept. 6 1906). Leyden's exhortation was met with tangible enthusiasm by the approximately 70 foreign participants and 350 German guests. Leo von Lewschin, director of the Moscow Cancer Institute, declared: “It is a great pleasure to see how different nations join forces in this question that affects humanity. I express hope that this conference, which is a milestone in the history of cancer research, will prove to be a blessing for suffering humanity” (Lewschin 1907). In his greeting, the director of the Imperial Cancer Research Fund, Ernest F. Bashford, welcomed this first conference as “the natural outcome of the Committee founded in Berlin in 1900″. “The truly international character of this conference”, he added, “is but a reproduction on a large scale of the international character of the Berlin committee itself from the time of its foundation” (Bashford 1907).

## International Association for Cancer Research 1908

**Fig. 2:**
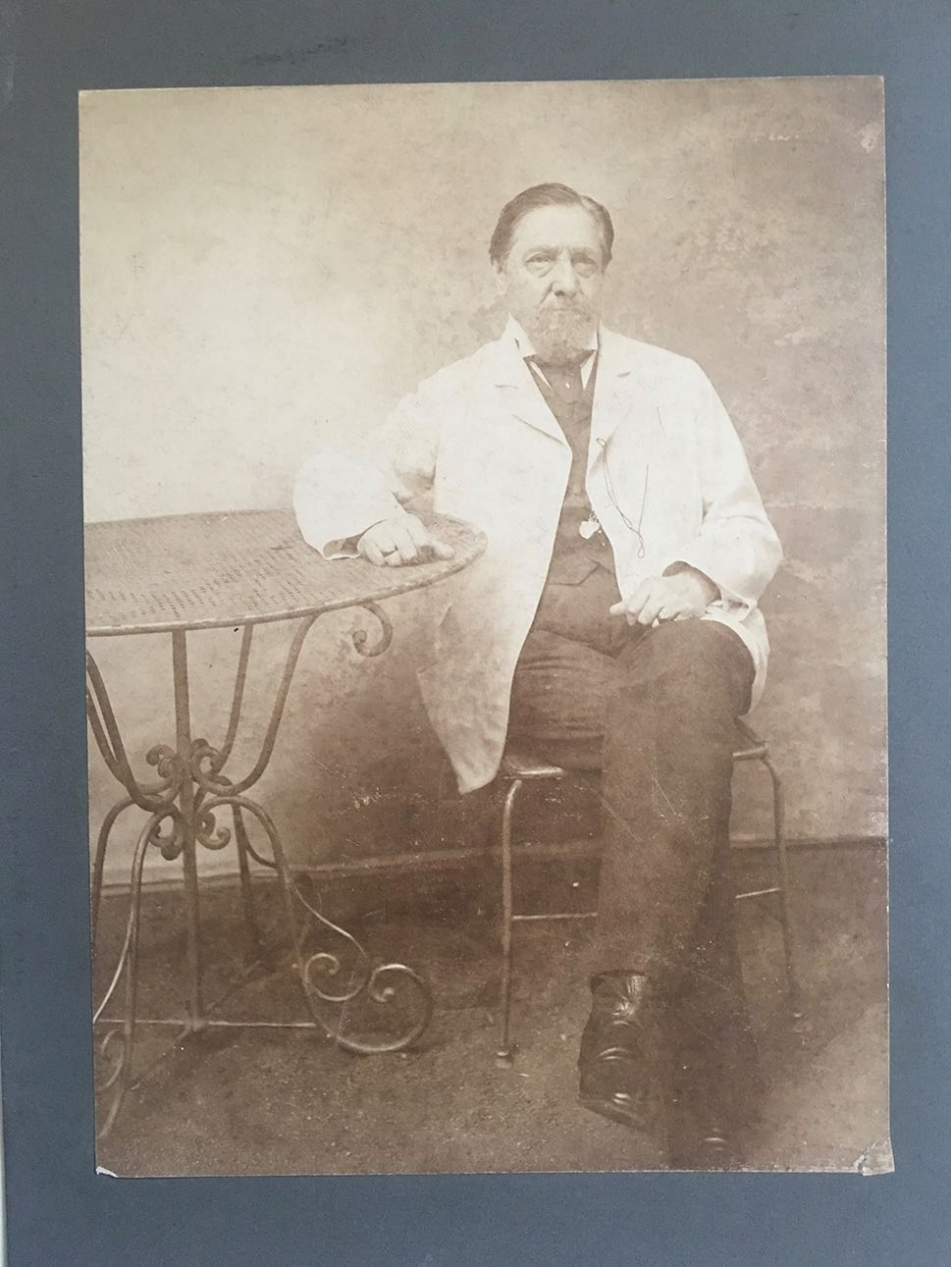
Professor Ernst von Leyden, Honorary President of the International Association of Cancer Research, around 1898. Courtesy of James von Leyden, Lewes/England

At the unanimous recommendation of the delegates, an International Association for Cancer Research was founded in Berlin only 2 years later, on May 23, 1908 (The Lancet 1908). Ernst von Leyden, then already past the age of 75, was its honorary president (Fig. 2). He had given ample proof of organizational talent with the founding of the sanatorium movement against tuberculosis, and of the "German Society for Internal Medicine" (1882); as an excellent clinician, he had treated emperors, kings and the Russian Czar Alexander III. But it was this last work, the masterful institutionalization of international cancer research in 1906 and 1908—against much resistance and national self-interest—that constitutes his enduring legacy. The International Association was then chaired by the Heidelberg surgeon Vincenz Czerny (Hansson and Tuffs 2016), who, two years earlier, had inaugurated his model cancer institute in Heidelberg in concomitance with the international conference. It should not be forgotten that during his trip to America in 1901 Czerny had gained important inspiration from Roswell Park and his research laboratory in Buffalo, New York. Now, on May 25, 1908, no less a personage than the German Chancellor, Prince Bernhard von Bülow, received the delegation of foreign guests, led by von Leyden and Czerny, including the representative of the newly founded American Association for Cancer Research, George Clowes of Buffalo. Two prominent follow-up congresses in Paris (1910) and Brussels (1913) brought together all the leading figures in cancer research, including James Ewing (New York), Roswell Park (Buffalo) and Gustave Roussy (Paris). These were by no means "occasional gatherings", as the Parisian oncologist and successor of Roussy [!], Pierre Denoix, would derogatorily put it seventy years later (Denoix 1982).

The reasons for the short lifespan of the so-called “I. Internationale” (Blumenthal 1928/1936) (First International Association) are to be found in political differences, unrealistic expectations and content-related aporia. "It was truly a tower of Babel", as the historian of science Alan Marcus recently put it (Marcus 2018). The professional approaches of the assembled surgeons, gynecologists, pathologists, epidemiologists, and practitioners were too different, and the national rivalries and ways of thinking were too pronounced. The platform of "cancer researchers", thrown together in this way, quickly came to an end in the carnage of the First World War. But what Marcus overlooked—his investigation period extended only to 1915!—was the fact that the transnational effort to fight against cancer actually survived and that after the war there were numerous clarification processes and new challenges. These culminated in the founding of a Second International Association in 1935, the *Union Internationale contre le Cancer* (UICC). Recent research has shown that its statutes (Paris 1935) were moulded on those of 1908 (Berlin) almost to the letter—although the political barycenter had shifted to Paris (Voswinckel 2019). An analysis of the lists of participants also shows that the efforts to revitalize the conferences (London 1928; Madrid 1933) involved quite a number of delegates who had already been in Heidelberg in 1906, e.g. the American William S. Bainbridge, the Englishman James Murray, the Frenchman Amédée Borrel and Ferdinand Blumenthal from Berlin (Voswinckel 2019).

## Historically inaccurate account of the founding

A hundred years later, the significance of Leyden's pioneering work is sadly unacknowledged, his role deliberately misrepresented, and his name erased from the international memory of today's oncology. The seven-part series “Landmarks in History of Oncology” by the New York pathologist Steven I. Hajdu relegates him to a mere footnote relating to a 1903 animal experiment, without even mentioning the 1908 International Congress (Hajdu and Darvishian 2013). In a rather casual list of specialist societies he compiled, the revitalized International Congress of 1933 (i.e.UICC) ranked solitaire between the Polish Anti-Cancer Society (1929) and the Connecticut Tumor Registry (1935) (Hajdu and Darvishian 2013). The Pulitzer Prize-winning study "The emperor of all maladies: a biography of cancer" by Siddhartha Mukherjee mentions neither one nor the other: Leyden’s name is nowhere to be found. The same applies to the 40-pages strong "Landmarks in Cancer Research 1907–2017″, celebrating the anniversary of the American Association of Cancer Research (AACR) (NN: Website of the AACR).

This may in part be because Leyden, as a cancer researcher in the narrower sense of the word, has left no lasting trace. His hope of having discovered a parasite as a cancer-causing agent proved to be an illusion and quickly became obsolete. The hypothesis of an infectious nature of cancer development, however, survived for a long time, in Paris as well as in Buffalo. If the American Association for Cancer Research (AACR) includes among the milestones the founding of scientific journals such as the Japanese journal "Gann" (1907), the Italian "Tumori" (1911) and the French "Bulletin de l'Association Francaise pour l'Étude du Cancer” (1911), then one might have expected a reference to the German “Zeitschrift für Krebsforschung” (1903). According to Michael Shimkin, this is the oldest cancer journal still in print, and was at the forefront of worldwide communication among cancer researchers at the time (Shimkin 1977). It was the then-president of the AACR, Erwin F. Smith, who in 1924, in the report of his European journey, described the “Zeitschrift für Krebsforschung” as “the leading cancer journal of the world” (Smith 1925). The founder and editor of this journal was none other than Ernst von Leyden.

## Two verdicts—inappropriate but effective

It is difficult to avoid the suspicion that this great initiator is the object to an unspoken prejudice. As we will show below, this prejudice has two roots, both largely determined by the political and ideological upheavals of the twentieth century: some saw Leyden as a typical representative of imperial Germany (which he was not); others thought he was too pro-Jewish (which was not untrue). The latter kind of racist remarks were sometimes heard in international oncology circles (see below).

Even in more recent Anglo-American and European literature, Leyden's role is distorted or marginalized (Pinell 1992). Alan Marcus cannot do better than resuming the distorted trope of the “imperial claim to leadership” being extended to the raising discipline of oncology after the rapid rise of the German Empire (Marcus 2018). Marcus makes no attempt to appreciate Leyden's personality, or frame him in a historical context: While other protagonists of his book are presented with biographical data (or at least the reader is directed to an obituary), Leyden’s persona is denied even this basic courtesy. In fact, all he has to offer is a congratulatory article on Leyden's 70th birthday in 1902 mistakenly cited as an obituary, to the effect that his activities between 1906 and 1908 are eclipsed. Yet, even that congratulatory article in question, appeared in JAMA, explicitly stated: “The occasion was taken as a suitable opportunity for felicitation by medical men all over the world. […] The spirit behind the work is an earnest of that better fellowship among the men of all nations that is so manifest and so promising at the beginning of the twentieth century and that has especially drawn the members of the medical profession together.”

It appears almost symbolic that, at the end of the twentieth century, von Leyden’s bronze statue at the Charité hospital was dismantled and moved to a cellar storeroom, after having being hit by a bullet in the head in the last days of the war in 1945. The 1996 demolition of the cancer barrack built by von Leyden in 1903—the core of the later cancer institute of the Charité—also went unnoticed by the oncological community (Voswinckel 2014).

## Perturbing and embarrassing fallacies

### How did it all come to this?

This question is answered in a monograph commissioned by the German Society for Hematology and Medical Oncology at the same time the monument was renovated (Voswinckel 2019). It reveals a historical lesson on the extent to which ideological excesses had a destructive impact on science and ruined the reputation of many German scholars.

All began with the exaggerated chauvinism and militarism of the imperial era, which expanded into a “war of the minds” (a technical term used by historians) (MacLeod 2014). The Germans' desire for hegemony, denounced everywhere by the Allies, led not only to the military defeat of the First World War but also to a later boycott of German science—and to a permanent loss of status for German as a language of science. In 1915, William Bainbridge (a participant in the cancer conference in Heidelberg 1906), visited in Berlin again on a semi-official mission and submitted a report to the American Senate on the German war aims. His report has been reprinted and translated many times, in the Petit Parisien and the Journal Belgique among others; it was reprinted in 1943 in the New York Times (New York Times 1943). The accompanying commentary asserts that “Time does not change the brutal German lust for conquest” and that” Time does not change the type of warped mentality that dreamed up the 'Master Race'“. Ernst von Leyden can easily be exonerated from such accusations of chauvinism. In Berlin, he was generally known for his worldly and liberal attitude; and he was also highly respected in Paris and Moscow.

At the time of his admission to the Académie des Sciences, Paris (1898) von Leyden was the only German physician member alongside Rudolf Virchow (Berliner Klinische Wochenschrift); in his capacity as treating physician of Czar Alexander III, he wore the Russian Order of Saint Anna (first class) with diamonds (Leyden and Lohde-Boettcher 1910). After 1933, the incriminated 'master race' with its aggressive anti-Semitism caused Jewish scientists to be expelled and deported; entire institutes (including the three cancer institutes in Berlin, Heidelberg and Frankfurt that existed in the German Reich) were closed, and there was a confrontation of “German science” with “Jewish science” at international congresses. Overnight, the person Ernst von Leyden, denounced as “Jewish”, was ostracized and became a “nonperson”. (Leyden was married in his second marriage to Marie von Oppenheim; his son was in a relationship with the Reichenheim family.) Leyden was now implicated with the fact that he had gathered so many successful Jewish students and assistants in his clinic (Klemperer, Blumenthal, Lazarus, Pappenheim, Michaelis, Fränkel). Due to a perfidious Nazi propaganda, all experimental cancer research carried out in Germany until then fell into the category of “Jewish pseudo-science”, so that the hard-liner “Reich German” cancer researchers were able to present themselves as an innovative vanguard force that swept out this science with a “new broom”.

Foreign visitors to congresses (Madrid 1933; Brussels 1936) had little choice but to conceal their amazement or look away. Through their pragmatic cooperation with their Reich German colleagues, they demonstrated a—from today's perspective—thoughtless and deceptive loyalty to the unjust regime. Only for a short time after the end of the war did the indignation about the German atrocities last: German and Japanese researchers were excluded from the first UICC post-war congress in St. Louis in 1947. But already at the successor congresses in Paris (1950), São Paulo (1954) and London (1958), one finds on all sides the willingness to cover up the ideological and political extremes of the past with a cloak of silence and to consign them to oblivion.

## Consequence of militarism, national socialism and racism

Let us remember: at the end of the war in 1945, the cancer research institute founded by Ernst von Leyden in 1903 was closed. Its expelled director Ferdinand Blumenthal was killed, his son, the lawyer and ministerial official Viktor von Leyden was in exile in India, his grandson Ernst von Leyden Jr. was shot dead by marauding soldiers in Berlin. In contrast, we find a new UICC President, Joseph Maisin, Brussels, whose sons had fought against the Germans; and cancer researchers like Georges Mathé, Paris, who as a student and resistance fighter had been deported to a concentration camp by the Germans (later founder of the European Society for Medical Oncology (ESMO); or the Belgian clinician Henri Tagnon, whose family twice had to flee from the German occupying forces (later founder of the European Organisation for Research and Treatment of Cancer (EORTC). Neither on an international level nor in Germany, which was henceforth divided into two parts, was there any reason to honor the memory of the German Ernst von Leyden and recall his importance for international oncology, as the center of cancer research increasingly moved towards the USA (Hansson et al. 2021) and English became the leading scientific language.

The situation was worsened by the fact that, after the loss of the cancer institutes in Berlin, Heidelberg and Frankfurt in the first post-war decades, Germany no longer had an intact organizational structure for oncology (as was the case in France and Belgium), but only solitary pathologists, surgeons or radiotherapists who were working in the fields of cancer research and therapy. Thus, research on the novel chemotherapy for the treatment of leukemias and lymphomas (mustard; aminopterin, etc.) in Germany was initially only taken up by well-positioned hematologists. Subsequently, hematologists claimed the sovereignty of interpretation over systemic cancer therapy for themselves, so that the treatment of solid tumors lagged behind. In addition, hematologists traditionally have a different approach to clinical routine and speak a different language than surgeons, gynecologists and radiotherapists, which made interdisciplinary communication difficult for a long time. It was not until clinical oncology was firmly established in the U.S. did internal medicine oncology become established in Germany under the influence of postdocs returning from the U.S. as the hub of interdisciplinary comprehensive cancer therapy. However, the close link between hematology and oncology was retained in the designation of the discipline and the professional association (German Society for Hematology and Medical Oncology (DGHO)).

How lastingly the continuity with Leyden's cancer research was interrupted, is revealed by the fact that at the 15th UICC Congress in Hamburg in 1990 (the first one on German territory!) not a word was spoken about Ernst von Leyden and the First International Congress. A myopic cultivation of tradition is also reflected in biographical reference works. For example, no mention is made of von Leyden in connection with the foundation of the “International Association for Cancer Research”, and also the names of his numerous, persecuted Jewish students (Klemperer, Blumenthal) are not mentioned. In retrospect, this looks very much like an attempt to obliterate and “undo” the anti-Semitic incidents of the past (Schadewaldt 1985). But how will a later generation of foreign specialist chroniclers, such as those mentioned above, be able to gain knowledge of relevant facts that have willfully been “ploughed under”?

The muddled and often broken history of the twentieth century cannot be embellished by even the most brilliant narratives. It requires a laborious work of recollection and reconstruction, which is simply not possible without confronting the sources. The historiography of oncology, with its different approaches to research in the English, French and American regions, is today more inconsistent than that almost any other field. A multilingual competence is required, in order to achieve a wide integrative account of international cooperation, based on historical and clinical expertise.

## The legacy of Ernst von Leyden

**Fig. 3:**
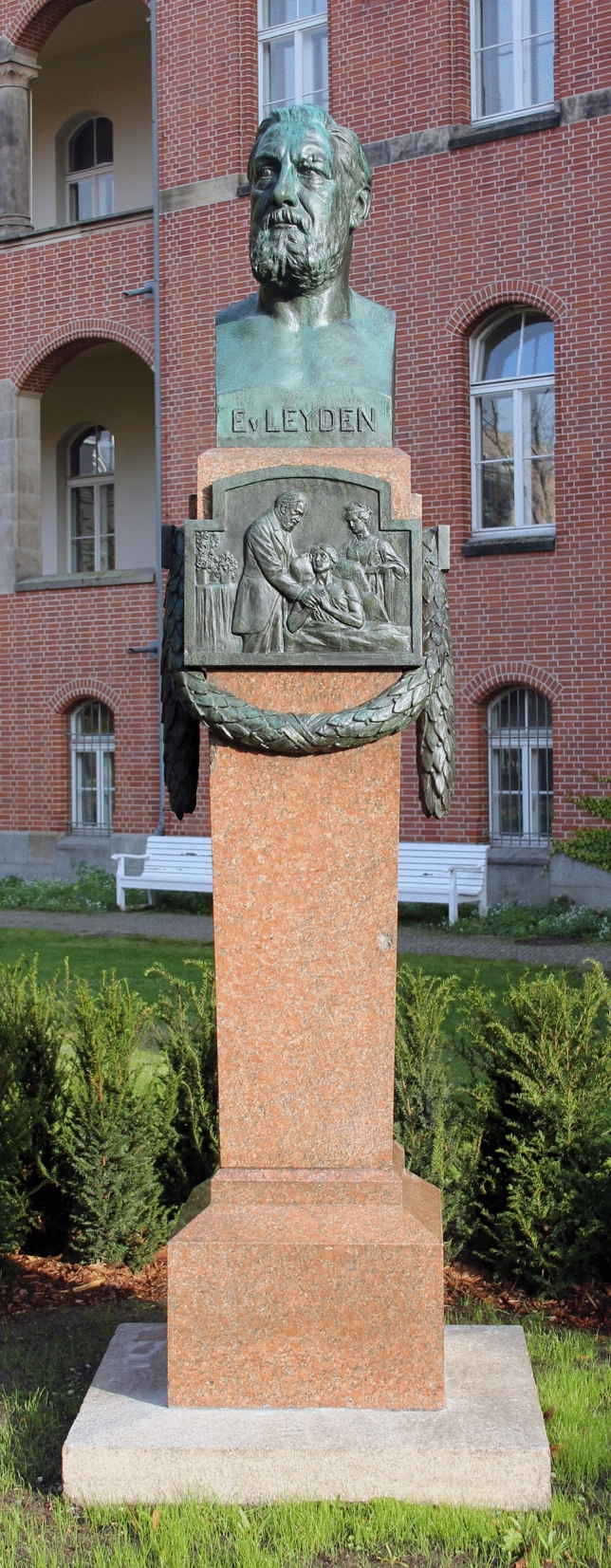
The renovated Leyden Monument of 1913 at the Charité, Berlin. Photo by OTFW, Berlin, CC BY-SA 3.0, https://commons.wikimedia.org/w/index.php?curid=83746309

In view of the resurgent anti-Semitism and nationalism everywhere, the German Society for Hematology and Medical Oncology (DGHO) is all the more determined to learn from past mistakes, to close the gaps in tradition, and to strengthen the self-image of international oncology. In October 2019, on the occasion of the DGHO's annual conference, the renovated Ernst von Leyden Monument from 1913 was reinstalled (Fig. 3), just a few minutes' walk from the “Berlin Medical History Museum of the Charité”, which hosts the specimen collection of the legendary Rudolf Virchow. The stately Leyden monument, with its height of 3.60 m, invites visitors from all over the world and especially oncologists from near and far to linger. It should be understood as.

a symbol of international cooperation and partnership,

as a memorial against national unilateralism and racist exclusion,

and as an incentive for mastering foreign languages.

## Data Availability

The datasets generated and analysed during the current study are available from the corresponding author on reasonable request.
